# At the Nexus between Cytoskeleton and Vacuole: How Plant Cytoskeletons Govern the Dynamics of Large Vacuoles

**DOI:** 10.3390/ijms24044143

**Published:** 2023-02-18

**Authors:** Hirotomo Takatsuka, Takumi Higaki, Masaki Ito

**Affiliations:** 1 School of Biological Science and Technology, College of Science and Engineering, Kanazawa University, Kakuma-machi, Kanazawa 920-1192, Japan; 2 Faculty of Advanced Science and Technology, Kumamoto University, Kurokami, Chuo-ku, Kumamoto 860-8555, Japan; 3 International Research Organization for Advanced Science and Technology, Kumamoto University, Kurokami, Chuo-ku, Kumamoto 860-8555, Japan

**Keywords:** vacuole, cytoskeleton, actin, microtubule, plant, development, environmental response

## Abstract

Large vacuoles are a predominant cell organelle throughout the plant body. They maximally account for over 90% of cell volume and generate turgor pressure that acts as a driving force of cell growth, which is essential for plant development. The plant vacuole also acts as a reservoir for sequestering waste products and apoptotic enzymes, thereby enabling plants to rapidly respond to fluctuating environments. Vacuoles undergo dynamic transformation through repeated enlargement, fusion, fragmentation, invagination, and constriction, eventually resulting in the typical 3-dimensional complex structure in each cell type. Previous studies have indicated that such dynamic transformations of plant vacuoles are governed by the plant cytoskeletons, which consist of F-actin and microtubules. However, the molecular mechanism of cytoskeleton-mediated vacuolar modifications remains largely unclear. Here we first review the behavior of cytoskeletons and vacuoles during plant development and in response to environmental stresses, and then introduce candidates that potentially play pivotal roles in the vacuole–cytoskeleton nexus. Finally, we discuss factors hampering the advances in this research field and their possible solutions using the currently available cutting-edge technologies.

## 1. Introduction

### 1.1. Cytoskeleton as a Rigid Yet Dynamic Intracellular Scaffold in Plant Cells

Cytoskeletons are essential elements for cellular function and viability, and constitute a system of filaments or fibers present in the cytoplasm of eukaryotic cells, with numerous functions, such as maintenance of cell shape, intracellular transport of vesicles and organelles, cell division, and cell signaling [1,2]. In animal cells, cytoskeletons are divided into three classes based on differences in size and protein composition. Microtubules (MTs), which are composed of the tubulin protein, are the largest type of filament, with a diameter of approximately 25 nm [3]. Dynein and kinesin act as motor proteins that transport cargo along the MTs, although there are no cytoplasmic dyneins in land plants [4]. The smallest type of cytoskeleton, i.e., actin filaments (F-actin), with a diameter of about 6 nm, are comprised of a protein called G (globular)-actin. F-actin is known to exert its function primarily in association with the myosin motor protein [3]. Intermediate filaments are named according to their sizes, that is, their diameter (~10 nm) is intermediate between that of F-actin and MT filaments [5]. Notably, the amino acid sequences of both tubulin and G-actin are highly similar between animals and plants, thus, the structures of plant F-actin and microtubules closely resemble those of animals [6,7]. The existence of plant intermediate filaments is still controversial [8].

Plant and animal cells greatly differ in their architecture due to the presence of rigid cell walls and large vacuoles. Accordingly, in addition to basic functions conserved in eukaryotes, plant cytoskeletons have evolved their unique features, which include acting as a template for cell wall construction and regulating the dynamics of large vacuoles that occupy the majority of the internal space of plant cells.

### 1.2. Large Vacuole, an Organelle with Versatile Functions

**Figure 1:**
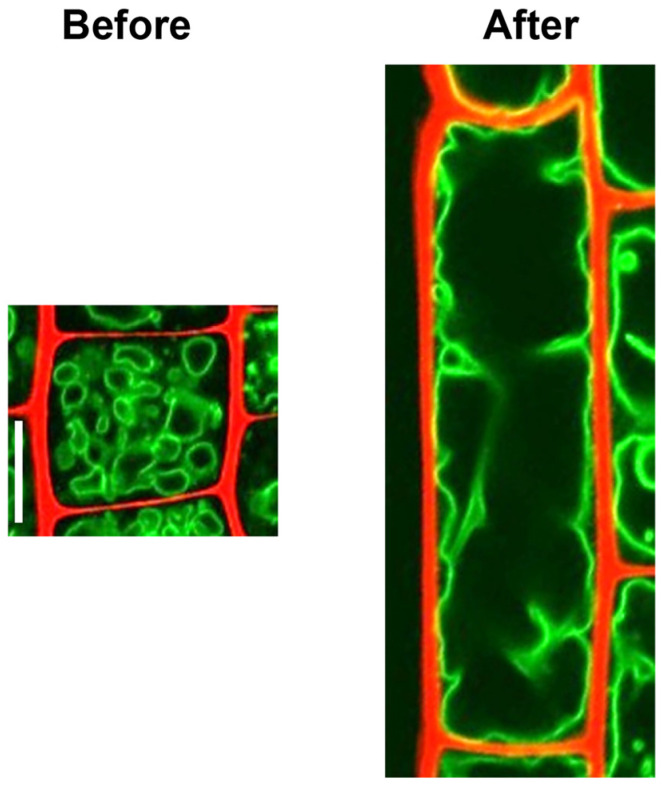
Vacuole status in root cells before and after cell enlargement. Confocal images of root epidermal cells in five-day-old *Arabidopsis thaliana* wild-type seedlings expressing tonoplast-localized GFP-VAM3 fusion protein under the control of *CaMV35S* promoter. The left panel represents a small, meristematic cell, while the right shows an enlarged, differentiated cell. Cell outlines are stained with propidium iodide (PI). The scale bar represents 10 µm.

Vacuoles are membrane-bound organelles filled with water containing inorganic and organic molecules, which form by fusion of multiple small vesicles, and are present in plant, animal, bacterial, fungal and certain protist cells [9]. The plant vacuole has numerous functions, such as water storage, isolation of toxic materials, pH maintenance, and autophagy [9]. In addition, during cell differentiation, vacuoles drive active plant cell growth by repeatedly fusing with each other and absorbing water to transform into large vacuoles that account for over 90% of the cell volume, which can generate turgor pressure exceeding 1 MPa (Figure 1) [9,10,11]. Vacuoles are surrounded by a lipid monolayer membrane and do not have any polarity; therefore, large vacuoles isolated from plant cells have been reported to be spherical [12]. Given the fact that large vacuoles affect the location of cell organelles, and that vacuolar rupture can lead to leakage of toxic compounds into the cytoplasm and subsequent immediate cell death, the dynamics of the large plant cell vacuoles must be strictly modulated by other cellular components, such as cytoskeletons. Extensive studies have shown that vacuolar size, number, and morphology are regulated in multiple ways, such as homotypic fusion, fragmentation, shrinkage, and invagination [13,14]. Dysregulation of vacuolar dynamics can impair plant development and viability, pointing to its control as being a pivotal factor for plants to successfully complete their life cycle and breed [15,16].

As sessile organisms, understanding the evolutionary strategy of plants in developmental processes and responses to environmental stimuli requires mandatory consideration of how vacuole dynamics are regulated by cytoskeletons. Nevertheless, only a limited number of cases demonstrating the roles of cytoskeletons in the regulation of vacuolar dynamics have been reported to date. In this review, we summarize previous findings and the unexplored gaps regarding cytoskeletal control of vacuolar dynamics in each developmental process and environmental response. Moreover, we identify the obstacles impeding the advances in this research field and discuss their potential solutions using the latest technologies.

## 2. Developmental Processes

Vigorous and sustainable plant growth is driven by spatiotemporally coordinated cell division and cell growth (cell expansion). One unique feature of plant cells is that even undifferentiated cells that are undergoing mitotic cell division carry large vacuoles, albeit to a lesser extent than the differentiated, expanded cells (Figure 1) [11]. Vacuoles can affect the positioning of organelles, including the nucleus, as physical obstacles; thus, precise control of vacuolar status is considered crucial for successful completion of mitotic cell division. Two modes of cell growth are found in plants, namely, diffuse growth and tip growth, both of which are common in that the formation of large vacuole serves as a driving force behind rapid expansion [17]. Unveiling how plant cells regulate vacuolar size and morphology along the differentiation trajectories that begin with cell division and end with cell expansion will provide fundamental clues for grasping the complete picture of plant growth and development.

### 2.1. Cell Division

**Figure 2:**
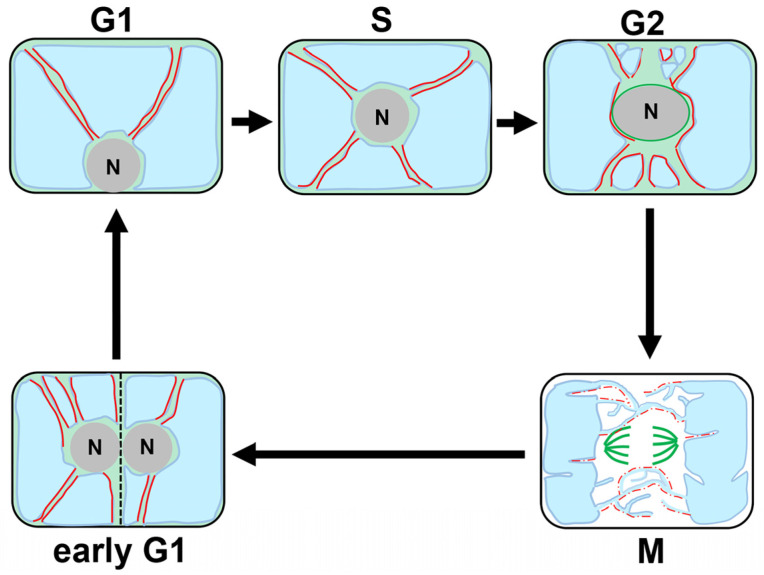
Model adapted from previous observations illustrating the reorganization of cytoskeletons and vacuoles during the cell cycle. Dynamic changes in the cytoskeletons and vacuoles during the cell cycle as revealed by studies using synchronized tobacco BY-2 cells. F-actin, MTs, vacuoles, and nuclei are shown in red, green, blue, and gray, respectively. Solid red lines represent F-actin running on the surface or in the vicinity of vacuole membranes, which is thought to be required for the TVS formation. Dotted red lines indicate F-actin supporting tubular vacuoles, which are expected to be present. Solid green lines in the G2 and M phase show MTs surrounding the nucleus and spindle MTs, respectively. F-actin around the nucleus and near the cell cortex is omitted in this model. MTs are observed inside the TVSs, but their localization near the vacuole membranes has not been determined; thus, the MTs in the TVSs are broadly shown in pale green.

Tobacco BY-2 cultured cells provide excellent cell cycle synchronization system, and have been used to reveal the 3D vacuolar dynamics during cell cycle progression, as illustrated in Figure 2: (1) During the G1/S phase, vacuoles are present as a compartmentalized entity, flanked by transvacuolar strands (TVSs) radiating from the nucleus to the cell periphery; (2) Cells at the G2 phase accumulate TVSs around the nucleus probably to facilitate the assembly of mitotic apparatus; (3) After entering the M phase, the TVSs converge on both sides of the future spindle, with newly formed tubular vacuoles termed “the tubular structure of vacuolar membrane (TVM)” encircling the mitotic spindle and eventually invading the region where the cell plate formation occurs (note that TVMs are not isolated compartments, but are rather extensions from the two large vacuoles flanking the mitotic apparatus and connecting them into one; and (4) After the telophase, when the TVMs between the daughter nucleus and the cell plate have eventually developed into a large vacuole, the TVSs emanating from the daughter nucleus toward the cell center are rapidly reconstructed at the early G1 phase (Figure 2) [18,19].

Time-sequencing observations combined with pharmacological interference of MTs or F-actin organization reveal that the dynamic vacuolar reorganization during the cell cycle is underpinned by the action of cytoskeletal elements (Figure 2) [18,20]. Once the F-actin, which is inherently present in the close proximity to the large vacuoles, is disrupted by an inhibitor of actin polymerization, the connectivity of the two large vacuoles via the TVMs breaks down, and the spherical vacuoles, instead of the TVMs, appear around the mitotic apparatus in BY-2 cells [20]. In addition, the absence of F-actin prevents TVS formation, eventually hampering nuclear migration toward the cell center at the early G1 phase [20]. Based on the findings by Higaki et al. [20], it can be speculated that F-actin together with myosin could significantly impact vacuolar dynamics in two ways: (1) F-actin on the surface or in close proximity to vacuole membrane physically supports the morphology and connectivity of the large vacuoles; and (2) actin bundles, initially running and swinging beneath the cell cortex, invaginate into the vacuolar lumen and shape TVS, which likely serve as a path for the nucleus to migrate toward the cell center at the early G1 phase [20].

Chemical disruption of MTs has demonstrated its participation in the TVM maintenance, though the MT organization in the vicinity of TVM is yet to be precisely determined [18].

The BY-2 tobacco cell cultures have been used to illuminate the dynamics of vacuoles and cytoskeletons during the cell cycle. Generally, cultured cells experience dynamic genomic reorganization prior to their establishment, and are maintained in the medium containing extremely high levels of phytohormones, which could confer them with altered mechanisms [21,22]. Therefore, it is crucial to verify that the principle described in this section is applicable irrespective of cell type.

### 2.2. Cell Growth

As mentioned above, plant cell growth is generally classified into two types, i.e., diffuse growth, which occur over a broader cell area, and the tip growth with restricted extension at the very apex of specialized cell areas [17]. Despite sharing common similarities, such as both requiring large vacuole formation to generate turgor pressure, the two modes of cell growth greatly differ in terms of cell structure, with diffusively growing cells displaying symmetric and uniform shape, whereas tip-growing cells displaying highly polarized and asymmetrical shape [17]; this review summarizes previous studies on each mode of cell growth in two separate sections.

#### 2.2.1. Diffusively Growing Cells

##### Root Cells

**Figure 3:**
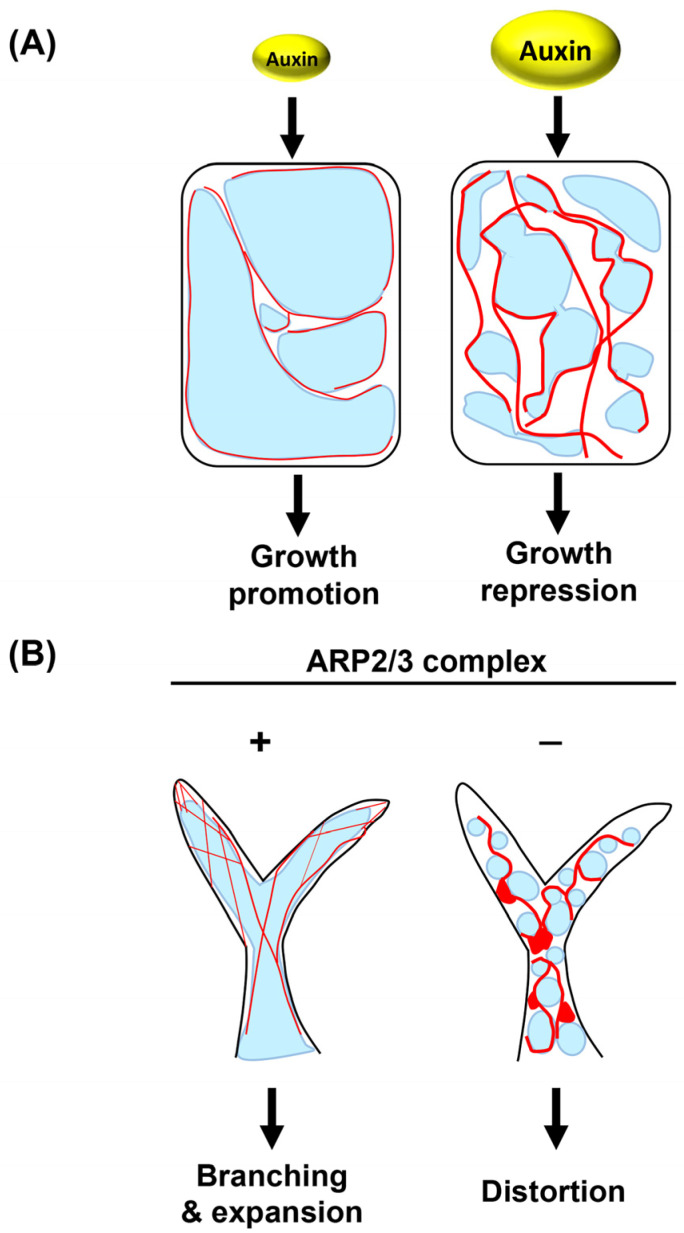
Schematic diagrams showing F-actin and vacuoles in diffusively growing root and trichome cells. (**A**) The F-actin is observed in the vicinity of large vacuoles in *Arabidopsis* root cells. An increase in auxin promotes actin bundling to evoke vacuole constriction, eventually leading to cell growth repression. (**B**) The thin F-actin running through trichomes is formed in an ARP2/3 complex-dependent manner in *Arabidopsis* developing trichomes. In the absence of the ARP2/3 complex, F-actin abnormally becomes thick and aggregates, likely resulting in small, spherical vacuoles. The F-actin and vacuole are shown in red and blue, respectively. Red distorted circles indicate F-actin aggregations.

Actin is at least one of the determinants of root cell vacuolar size, as evidenced by the finding that pharmacologically induced disruption or stabilization of F-actin can lead to abnormally small or enlarged vacuoles, respectively [23]. Presumably through this function, actin plays a decisive role in accelerating diffusive cell growth in roots [24]. Intriguingly, the dual-labeling assay has identified potential actin–vacuole contact sites in diffusively growing root cells of *Arabidopsis thaliana* (Figure 3A) [23], suggesting the possibility that F-actin physically supports vacuolar enlargement through water uptake. In stark contrast, MTs are barely detected in the proximity of vacuolar membranes at this stage, which implies that MTs are not the primary factor controlling vacuolar dynamics during the diffuse growth [23]. This view was supported by the observation that disruption of MTs by oryzalin showed no significant impact on vacuolar morphology [23].

Auxin is a phytohormone that governs a plethora of cellular processes in plants, including a repressive role in root cell elongation, and has been shown to be an upstream regulator of actin organization in roots [23,25,26]. Previous reports have indicated that long-term continuous exposure to exogenous auxin can promote actin bundling in roots (Figure 3A), while short-term treatment unbundled the F-actin within minutes [23,25,26]. Scheuring et al. showed that exogenous auxin application generated vacuoles that appeared small and numerous but were actually interconnected (Figure 3A) [23], implying the role of auxin in altering vacuolar morphology by constriction rather than by fragmentation. Since it has been demonstrated that auxin is capable of stabilizing some members of vacuolar soluble N-ethylmaleimide-sensitive-factor attachment protein receptor (SNARE) proteins, such as VESICLE-ASSOCIATED MEMBRANE PROTEIN 711 (VAMP711), SYNTAXIN OF PLANTS 21 (SYP21), and SYP22, thereby playing a role in determining the roots vacuole morphology, the presence of actin-independent pathways modulating vacuolar morphology and size cannot be ruled out [27]. Nonetheless, given that both actin and myosin mutants are partially insensitive to changes in vacuolar structures induced by either an increase or decrease in auxin levels, it is plausible that auxin exerts its impact on vacuole morphology at least partly through the actin–myosin system [23].

##### Trichome

Trichomes are epidermal cell outgrowths on leaves, which are implicated in a broad range of biological processes, such as defense against phytophagous insects and UV protection [28]. Despite its polarized shape with typically three branches, the mode of trichome cell growth is classified as diffuse growth, because it not only grows at the apical end, but also in the shank and at the basal region [29].

Trichome morphogenesis is governed by the ARP2/3 complex, which is an important regulator of actin organization in a variety of species, and the absence of an intact ARP2/3 complex has been shown to cause swollen and twisted trichomes with decreased branch length in *Arabidopsis* [30,31]. In contrast to thin F-actin running through trichomes of the wild type, those lacking the ARP2/3 complex displayed aggregated actin arrays in more random orientations (Figure 3B) [31]. This defect is likely responsible for transforming the vacuoles, which would otherwise be large and singular, into smaller and spherical trichomes of ARP2/3 mutants (Figure 3B). These findings suggest that, similar to the root cells, properly organized actin is required for vacuole enlargement [31].

Based on these findings, it is reasonable to conclude that actin plays a role in supporting vacuolar enlargement in diffusively growing plant cells. It should, however, be emphasized that the role of actin in vacuolar dynamics has not been investigated in diffusively growing cells other than roots and trichome cells. Thus, it will be of importance to examine whether the principle found in the root epidermis and trichome holds true for other types of diffusively growing cells.

#### 2.2.2. Tip Growing and Tip Growing-like Cells

The tip growth is a mode of cell growth that causes cells to elongate only at the very tip of the specialized area, leading to a cylindrical protrusion represented by pollen tubes and root hairs in plants [32,33,34,35]. The prevailing view is that the tip-focused actin is crucial for tip growth, while the primary driving force of diffuse growth is turgor pressure derived from vacuoles. Nonetheless, large vacuoles extend nearly to the tip of pollen tubes and root hairs, prompting the speculation on vacuolar involvement in the tip growth. Indeed, the necessity to control vacuolar dynamics for tip growth has been gradually uncovered through years of challenges.

##### Pollen Tube

Pollen tubes are the most extensively studied model of tip growth by virtue of their ease of observation and rapid growth. For example, a previous study demonstrated that the lily pollen tubes grew as fast as 18 µm/min, which is one of the fastest rates of cell growth in plants [36]. In angiosperms, the pollen tube, bearing two sperm cells and one vegetative cell nucleus within it, is expected to deliver sperm cells to the egg and central cells as rapidly as possible [37]. To enable this, pollen tubes elongate actively with tip growth.

**Figure 4:**
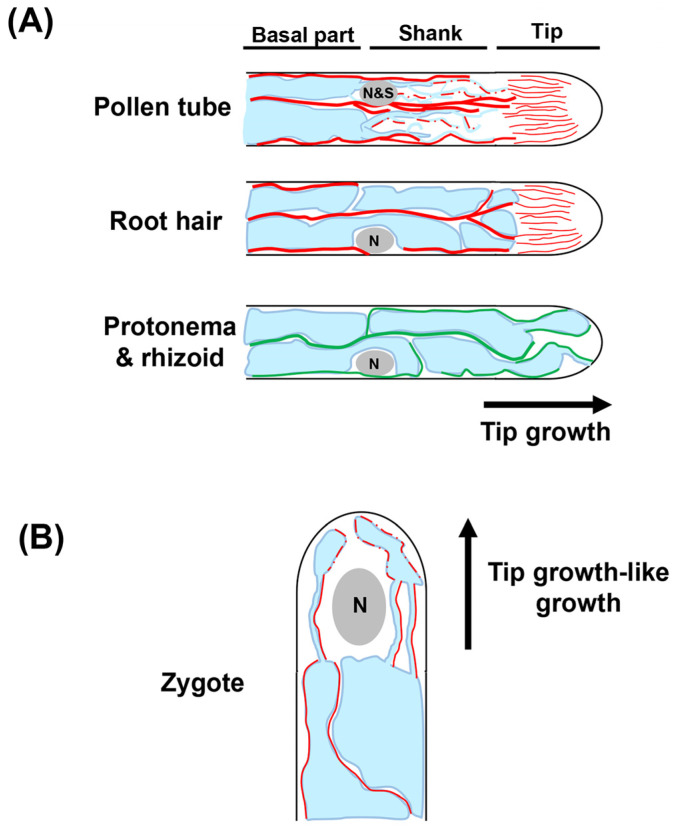
Schematic diagram showing vacuolar structures and the adjacent cytoskeletons in tip-growing and tip-growing-like cells. (**A**) Tip-growing region of a pollen tube and root hair of *Arabidopsis* as well as of protonema and rhizoid of *Physcomitrium patens*. The MTs of pollen tube/root hair and F-actin of protonema/rhizoid are not indicated in the schematic since their role in vacuole formation is still obscure. Thick and thin solid red lines indicate thick actin bundles and thin actin filaments, respectively. Dotted red lines in pollen tube show F-actin that is thought to be present on the surface of tubular vacuoles. (**B**) A growing zygote of *Arabidopsis*. F-actin, MTs, vacuoles, and nuclei (and sperm cells in pollen tube) are shown in red, green, blue, and gray, respectively. Solid red lines indicate F-actin observed in the proximity of large vacuoles and tubular vacuoles, while dotted red lines show F-actin in the apical region, which has not been precisely determined.

Pollen tubes display a highly polarized vacuolar structure while undergoing tip growth. During early pollen germination, numerous small vacuoles spread from the pollen grain toward the elongating tube [38]. At the late stage, the extreme tip of the pollen tube is devoid of large organelles, including vacuoles, whereas fine, thread-shaped vacuolar strands are observed in the shank of the pollen tube (Figure 4A). Moreover, the more basal part of the pollen tube is occupied by a large vacuole (Figure 4A) [38,39,40].

F-actin, as well as vacuoles, is organized with polarity; that is, long and thick actin cables, which act as scaffold for cytoplasmic streaming, run through the shank, whereas the subapical and apical areas are rich in fine F-actin (Figure 4A) [35]. In the presence of an actin polymerization inhibitor latrunculin B (LatB), large vacuoles penetrate into the apex of the pollen tube, while thread-like vacuoles in the shank are abnormally aggregated [41]. These findings led to the as-yet experimentally unproven hypothesis that zone-specific vacuole morphology is determined by differential actin organization in pollen tubes.

A previous study showed that the disruption of MTs by oryzalin did not cause any obvious defects in vacuolar status, suggesting that actin, rather than MTs, is primarily engaged in the maintenance of vacuolar morphology and distribution in pollen tubes [41]. However, a more recent report indicated that MTs are required for transporting prevacuolar compartments to large vacuoles in the shank of tobacco pollen tubes, which points to the possibility of MTs engagement in fine-tuning vacuolar dynamics [42].

The findings described in this section were mainly achieved using lily flower pollen, which has been widely used to study the growth kinetics of pollen tubes due to its rapid elongation property. A similar polarized distribution of vacuoles has been observed in other species, such as *Arabidopsis* and tobacco [16,42,43], implying that the homologous mechanism controlling vacuole dynamics might be conserved in angiosperms.

##### Root Hair

Growing root hairs display the following cellular structural similarities to pollen tubes: (i) the extreme apex is rich in cytoplasm with a lot of vesicles; (ii) the subapical area contains components that are required for cell expansion, including the nucleus, fine F-actin, and MTs; and (iii) the large vacuole occupies the shank for turgor pressure generation that is required for tip growth, with thick actin bundles as well as MTs penetrating the shank (Figure 4A) [33,44]. Intriguingly, thread-like vacuolar strands, which are observed in pollen tubes, are not formed in root hairs, demonstrating that the intracellular architecture of pollen tubes and root hairs are closely similar but not identical (Figure 4A) [45].

Among numerous mutants with impaired root hair elongation phenotype, the phenotype of *root hair defective 3* (*rhd3*) mutant has been implicated in altered vacuolar formation [45]. It has been shown that the *RHD3* gene, which encodes an evolutionarily conserved dynamin-related GTPase, is involved in the morphological control of endoplasmic reticulum and perhaps thereby promotes vacuolar fusion [46]. In the absence of the functional *RHD3* gene, root hairs display a striking reduction in vacuolar size, indicating that large vacuolar formation is associated with root hair elongation [45].

Cytoplasmic streaming through TVSs penetrating the shank of root hairs is considered vital for the delivery of molecules to the tip of root hairs. The essential role of F-actin in the maintenance of TVS in root hairs has previously been demonstrated [47]. Microinjection of antiserum against villin protein involved in actin bundling was shown to transform the thick F-actin penetrating the transvacuolar strands into thin filaments [47], which might have resulted in the disappearance of TVS and altered direction of cytoplasmic streaming [47]. Combined with the case of dividing cells and pollen tubes described above, thick F-actin might generally serve as physical elements that support TVSs (Figure 2 and Figure 4A).

Despite extensive studies on root hair growth, the molecular mechanism by which the cytoskeletons form zone-specific vacuolar structures remain elusive, and its elucidation will accelerate the understanding of still enigmatic optimization processes of plant root hair lengths in response to developmental and environmental stimuli.

##### Zygote

After fertilization, zygotes of flowering plants elongate into the apical–basal axis in a highly polarized manner reminiscent of tip growth, and place their nucleus at the apical tip [48,49]. This polarized nuclear positioning has been shown to be essential for the first asymmetric division producing small apical and large basal cells, with the former giving rise to the embryo [50], showing that the polar cell growth of zygotes is the very first step of plant development.

In most flowering plants, including *Arabidopsis*, the unfertilized egg cell is predominantly occupied by the large vacuole. Upon completion of fertilization, the large vacuole shrinks and/or disperses within the zygote, and thin tubular vacuoles are subsequently formed around the nucleus (Figure 4B) [51]. The vacuoles then accumulate predominantly at the basal region as the zygote elongates (Figure 4B). The uneven vacuolar distribution ensures polar nuclear localization at the apical area of the zygote, thus contributing to asymmetric zygote cell division. In the *shoot gravitropism2* (*sgr2*) mutant, which develops large vacuoles not only at the basal region, but also at the apical area, the nuclear migration toward the apical end is not feasible, resulting in a more symmetric zygote division [51]. A recent study discovered that the F-actin run along the vacuole membranes, and that treatment with actin polymerization inhibitors, such as LatB and cytochalasin D (CytD) disrupted the perinuclear tubular vacuoles in zygotes (Figure 4B) [50]. These findings imply the roles of F-actin in the establishment and maintenance of vacuolar structures within zygotes.

##### Protonema and Rhizoid in Moss

Moss generally harbors two cell types that undergo tip growth, namely, protonema apical cells and rhizoid apical cells, with the former as the most apical cell in thread-like cell chains that form the earliest stage of gametophyte development during the moss life cycle. In contrast, the rhizoid apical cells are similar to root hairs in both morphology and physiological functions [52,53].

Moss cells undergoing tip growth are similar to the tip-growing cells of flowering plants in that both harbor a large vacuole. However, they differ in at least two respects. First, the protrusions from the large vacuole repeatedly contract and extend toward the apex of rhizoids in *Physcomitrium patens*, which is in clear contrast to the pollen tubes and root hairs of flowering plants with the apex devoid of large vacuoles (Figure 4A) [54]. Second, vacuolar morphology in tip-growing cells of moss is controlled by MTs rather than actin (Figure 4A). Upon depolymerization of MTs in the protonema apical cells of *Physcomitrium patens*, the apical tubular vacuoles turn spherical, and the cell apex aberrantly accumulate cytoplasm [54]. Moreover, vacuolar protrusions, which repeat dynamic movement toward the cell apex under normal conditions, are rapidly lost in rhizoids treated with oryzalin [54]. Notably, MTs are located near large vacuoles and in the TVSs, further supporting the notion that the MTs are engaged in vacuole morphology in moss (Figure 4A) [54]. In contrast, the application of the actin depolymerization reagent showed no obvious impact on the vacuolar states in *Physcomitrium patens* [54]. Taking into account that vacuole dynamics are mainly regulated by actin in angiosperms, as described above, these findings suggest that the mechanism underlying cytoskeletal control of vacuolar dynamics has diverged during the process of land plant evolution. Tracing the time when, and the reason each plant species acquired its own mechanism controlling their own vacuolar dynamics should be of particular interest. In addition, structures of large vacuoles greatly differ among cell types, such as pollen tube, root hair, and protonema/rhizoid, as summarized in this section. This suggests that vacuolar dynamics are not per se critical determinants that confer the capability of tip growth (Figure 4A).

## 3. Responses to Environmental Stimuli

The large vacuole serves not only as an important regulator of cell shape and size, but also as a reservoir of enzymes that trigger programmed cell death (PCD); thus, vacuolar dynamics require precise control in response to fluctuating environments.

### 3.1. Stomatal Opening and Closure

**Figure 5:**
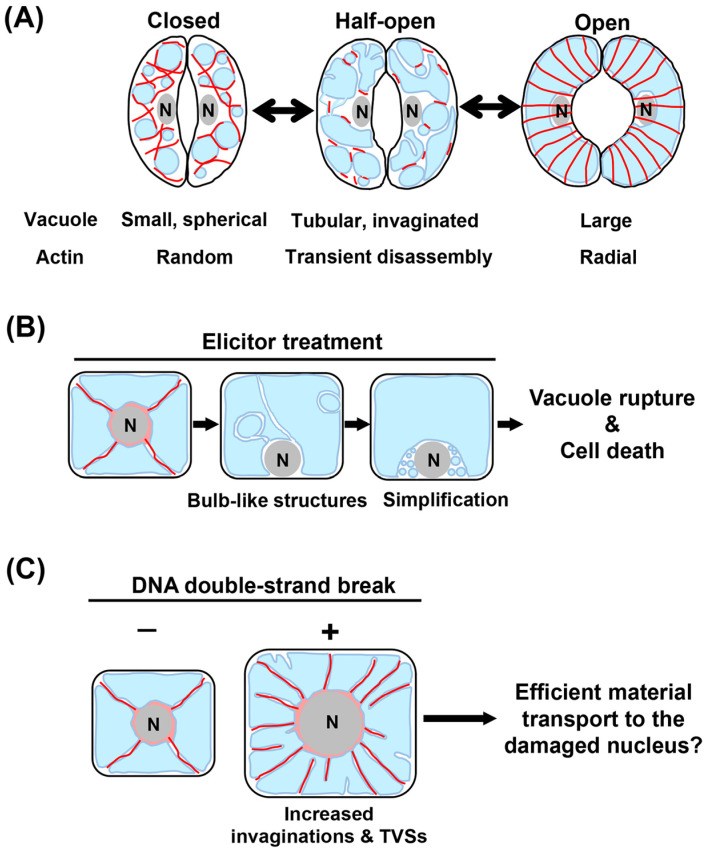
Dynamics of vacuoles and/or actin filaments during responses to environmental stimuli. (**A**) A possible model illustrating dynamics of F-actin and large vacuoles during stomatal movement in *V. faba*, *Arabidopsis*, and tobacco. (**B**) Elicitor-induced changes in vacuolar structures in BY-2 cells. Actin behavior upon elicitor treatment is yet to be determined. (**C**) The status of F-actin and vacuoles in DNA-double strand break-suffering BY-2 cells. F-actin, vacuoles, and nuclei are shown in red, blue, and gray, respectively.

Stomata consist of paired guard cells that repeatedly open and close at high speed for gas exchange and transpiration in response to changes in environmental factors associated with carbon assimilation and water loss [55,56]. The stomatal aperture is primarily governed by the turgor pressure generated by vacuoles in each guard cell, and stomata alternatingly open with increased vacuolar volume in the guard cell and close as the vacuolar volume decrease (Figure 5A) [57]. When closed stomata open, small and spherical vacuoles become enlarged via the intermediate state with tubular vacuoles and bulb-like intravacuolar structures, and vice versa when open stomata close (Figure 5A) [57]. Interestingly, this dynamic reorganization of vacuolar structures can be completed as rapidly as within 30 min [58].

In addition to the regulatory roles of Ca^2+^ channel at the plasma membrane, F-actin has crucial functions in modulating vacuole morphology, thereby optimizing stomata aperture [57,59]. In various plant species, such as *Vicia faba*, closed guard cells exhibit randomly oriented F-actin, while open guard cells harbor radial F-actin (Figure 5A) [60,61,62,63]. Interestingly, in *V. faba*, *Arabidopsis*, and tobacco, temporal disassembly of F-actin is commonly observed during both stomatal closure and opening (Figure 5A) [57,59]. Since the pharmacological stabilization of F-actin retards stomatal opening, the transient disassembly of long F-actin is likely one of the critical steps for rapid stomatal movement [59,64]. It is noteworthy, however, that similar transient actin disassembly unlikely occurred during *Arabidopsis* stomatal movement in response to diurnal cycles [65]. Careful examination across species under various environmental conditions will uncover the physiological significance of transient actin disassembly in stomata movement.

Closer observations have revealed that F-actin surround small, unfused vacuoles in closed stomata, and that fusion of small vacuoles begins concurrently with the depolymerization of F-actin at the onset of stomatal opening (Figure 5A) [57,59]. These findings suggest that F-actin serves as a physical obstacle inhibiting vacuolar fusion during stomata opening. On the other hand, since the actin rings are formed at the contact site of fusing vacuoles, it is also plausible that F-actin is required for vacuole fusion [59]. Given these observations, it is likely that, during stomatal movement, actin plays antagonistic roles in the regulation of vacuole structures by inhibiting and promoting vacuole fusion. To the best of our knowledge, however, no attempts have been made to clarify the molecular basis of how actin accomplishes its dual roles. Similar to observations in F-actin, MTs have been reported to exhibit regular patterns during stomatal movement [66,67]. Yet, the role of MTs in vacuolar dynamics is still poorly defined, which warrants future investigations.

### 3.2. Elicitor-Induced Cell Cycle Arrest and PCD

Upon exposure to abiotic stresses, plant cells evoke various types of cellular responses, of which the most drastic are cell cycle arrest and PCD [68,69]. Vacuoles, which store enzymes possessing caspase-like activities, are the key to triggering PCD [70,71]. For example, vacuolar rupture induced by tobacco mosaic virus causes vacuole-localized proteases with caspase-1-like activity to leak into the cytoplasm, eventually evoking PCD in tobacco leaves [72]. Thus, unraveling the behavior of cytoskeletons and their roles in maintaining vacuolar integrity will significantly enhance the understanding of how plants cope with pathogen attacks at the cellular level.

An elicitor protein, cryptogein, secreted by phytopathogenic pseudo-fungus *Phytophthora cryptogea*, has been reported to induce cell cycle arrest and ultimately PCD [73,74]. A study using tobacco BY-2 cultured cells demonstrated that exposure to cryptogein at the S or M phase arrested the cell cycle progression at the G2 or G1 phase, respectively, followed by PCD [75,76,77]. Upon cryptogein-induced cell cycle arrest, vacuolar morphology was reported to rapidly reorganize in the order described below: (1) the TVSs emanating from the nucleus toward the cell periphery rapidly disappeared; (2) the bulb like intravacuolar structures formed inside the large vacuole; (3) overall vacuole structures were simplified, with the bulb like structures disappearing; and (4) the large vacuole ruptured, causing cell death (Figure 5B) [76]. Pharmacological interference with actin polymerization accelerates the disappearance of bulb-like structure and vacuolar rupture, implying the possibility that F-actin acts as a safeguard and physically maintains the integrity of vacuolar membranes against undesirable cell death caused by accidental vacuolar rupture [76]. Future determination of the dynamics of F-actin in the vicinity of vacuolar membranes will further reinforce this concept.

### 3.3. DNA Damage-Triggered Cell Growth

Genotoxic stress damages the genomic DNA leading to harmful mutations, and is one of the most deleterious plant stresses caused by environmental stressors [78]. To reduce the risk caused by propagation of mutated cells, and increase cell volume to ensure organ growth, plant cells with DNA-double strand breaks (DSBs) cease mitotic cell cycle and instead undergo endoreplication, which can induce dramatic cell enlargement by increasing DNA content through repeated DNA replication without cytokinesis or mitosis (Figure 5C) [79,80]. A previous report found that induction of endoreplication by zeocin, an inducer of DSBs not only enlarged vacuoles, but also caused invagination, which eventually resulted in TVSs that were likely connect to the nucleus in BY-2 cells (Figure 5C) [81]. To repair DSBs, the nucleus necessitates many factors, including DNA repair proteins, nucleotides, and ATP. Due to the above observations, a possible explanation for this intriguing response is that the TVSs generated by vacuolar invaginations serve as routes for materials required to repair DSB in the damaged nucleus. This view is supported by the finding that physical disruption through laser ablation of TVSs, which harbor fine F-actin inside them, can stop the movement of cytoplasmic substances, such as mitochondria [81].

## 4. Candidates Controlling Vacuolar Dynamics at the Nexus between Cytoskeletons and Vacuoles

From our discussion so far, it is evident that actin potentially play a dominant role in controlling vacuolar dynamics in angiosperms. This view has been supported by proteomic analysis using *Arabidopsis* seedlings, which detected a direct interaction between G-actin rather than tubulin and plant vacuoles [82]. However, it currently remains ambiguous whether, and if so, how, filamentous actin co-localizes with vacuoles to modulate vacuolar shape. As described in this review, actin seems to have both promotive and inhibitory roles in large vacuolar formation; therefore, analyzing the behaviors of F-actin or vacuoles alone is insufficient to understand how actin exerts its action on vacuolar dynamics. Consequently, it is essential to identify actin-associated molecules that confer mobility, polarity, and complexity to the vacuoles at the nexus between vacuolar membranes and F-actin. Previous studies have identified some candidates as a linker between F-actin and vacuoles. The possible roles of such candidates with reference to their corresponding roles in animals and yeasts are discussed below.

### 4.1. Myosin

Myosin has a vital role in controlling vacuolar morphology, as indicated by the following findings: (1) During the mitotic cell cycle, treatment with the general myosin ATPase inhibitor, 2,3-butanedion monoxime (BDM), could hampered vacuolar reorganization [20]; (2) Myosin mutants, such as *xi-k/1/2 and xi-k/1/2/i*, exhibited partial resistance to auxin-induced vacuolar constriction in root cells [23]; (3) Artificial induction of vacuolar deformation using optical tweezers required a higher force in BDM-treated cells [83]; and (4) Biochemical analyses have isolated several myosin-related proteins that interact with plant vacuoles [82]. The molecular basis of myosin-driven vacuolar reorganization has been clearly demonstrated in the budding yeast. Briefly, the class V myosin Myo2p directly binds to vacuole-specific receptor Vac17p through its cargo-binding domain, and then, directionally moves along the actin cable during cell division, thus ensuring that the vacuole is inherited two daughter cells [84,85,86]. In light of this, one possible explanation is that myosin might contribute to vacuolar dynamics by moving on actin fibers with a small vacuole in plant cells. To date, however, the determination of how myosin, together with actin, modulate vacuolar size and shape at the molecular level in plants is yet to be reported.

### 4.2. ARP2/3 Complex

The ARP2/3 complex consists of seven proteins that initiate actin polymerization and branching in eukaryotic cells, and it represent another promising candidate that connect actin and the plant cell vacuole [87,88]. As described earlier, the ARP2/3 complex is crucial in large vacuole formation in trichomes. Similarly, a recent study reported that the ARP2/3 complex is required for vacuolar fusion in leaf pavement cells [87,89]. In both cases, it is yet to be examined whether the ARP2/3 complex acts in close proximity to the vacuolar membrane or if it remotely affects vacuolar morphology by providing the actin organization required for vacuolar fusion. In budding yeast, the absence of an intact ARP2/3 complex was shown to cause abnormally fragmented vacuoles, indicating a similar function of the yeast ARP2/3 complex in vacuolar fusion [90]. Remarkably, proteomic analysis using purified vacuoles from yeast cells yielded not only actin, but also the ARP2/3 component, which suggested that the ARP2/3 complex could govern vacuolar dynamics by controlling actin organization on the vacuolar membrane [90]. In contrast, no direct interactions between the ARP2/3-related factors and vacuolar membranes have been reported in plants, and would be resolved by higher-resolution imaging of subcellular localization of the ARP2/3 complex.

### 4.3. The Adaptor Protein (AP) 3 Complex

AP complexes are heterotetrameric proteins, which mediate intracellular membrane trafficking along endocytic and secretory transport pathways [91]. AP complexes occur in five different variants designated AP1 to AP5, each of which displays distinct subcellular localization, and distinct roles in post-Golgi trafficking [91]. AP3 complex is implicated in vacuolar dynamics in plants; it has been shown to facilitate cargo trafficking from the Golgi body to the vacuoles, thereby controlling morphology of large vacuoles [92]. Notably, the medium subunit of the AP3 complex (AP3M) has the ability to directly bind to and sever F-actin. This might suggest that the AP3 complex localizes at the vacuole–actin nexus through AP3M to modulate vacuolar morphology. Similar actin-binding activity was detected in the mouse AP3M, which implies that the AP3M function is conserved in both plants and animals [92]. Moreover, the yeast AP3 complex has been demonstrated to be involved in the selective cargo transport to the vacuoles [93]. Thus, it is likely that the AP3 complex contributes to vacuolar formation in a manner conserved throughout eukaryotes, rather than in a plant-specific manner.

### 4.4. NET4

The candidates introduced so far are widely conserved in plants and animals; however, given that large vacuoles are structures that characterize the unique behavior of plant cells, it is reasonable to predict that plants have their specific factors that link F-actin and large vacuoles. The plant-specific actin-binding protein NETWORKED family carries not only an actin-binding domain, but also a membrane-associated domain, which make them an attractive candidate that acts at the nexus between F-actin and membrane-bound organelles [94]. Of the 13 reported NET family protein members, NET4A and NET4B have been implicated in the control of vacuolar morphology. Both overaccumulation of NET4A, and the simultaneous absence of NET4A and its closest homolog NET4B, resulted in a spherical, large vacuoles in root cells, which resembled the vacuole shapes in cells with either abnormally stabilized or destabilized F-actin [95]. The GFP-fused NET4A protein showed both localization to the F-actin and tonoplast membrane, and the protein was also highly accumulated at the constricted vacuolar membranes, which suggested that NET4s are likely to optimize vacuolar compactness through constriction at the vacuole–actin nexus [95]. It will be crucial to explore other unique plant factors, and to clarify how they, including NET4s, regulate vacuolar dynamics at the F-actin and vacuolar nexus.

## 5. Conclusions and Perspectives

Despite extensive previous studies, our understanding of the molecular mechanism underlying cytoskeletal control of vacuolar dynamics remains far from complete. Here, we discuss the challenges in studying vacuole and cytoskeletons and their potential solutions for future research.

### 5.1. Strategies for Studying the Roles of Vacuoles and Cytoskeletons

One of the major obstacles hampering advances in this research field is the indispensable role played by vacuoles and cytoskeletons throughout the plant life cycle. Myriad factors controlling cytoskeleton organization and/or vacuolar dynamics have so far been identified. Importantly, however, since both the vacuole and cytoskeletons play essential roles in almost all cell types in plants, many mutant cells lacking these factors display early lethality or severe growth defects that make it impossible to analyze their roles in specific cell types, tissues, or organs of interest. For example, the *vacuoleless1* mutant, with severe defects in the formation of large vacuoles, exhibits gametophytic and embryonic lethality, and thus cannot be employed to study the significance of large vacuole formation in post-embryonic development [15,38]. One effective strategy for solving this problem is to identify chemical compounds that block the specific pathway of vacuolar or cytoskeleton formation. A typical example is the recent chemical screening and the discovery of chemical compound disrupting vesicle trafficking to the vacuole, which is regulated by essential v-SNARE proteins, VESICLE TRANSPORT V-SNARE 11 (VTI11) and VTI12 [11]. This chemical was used to reveal the contribution of endocytic trafficking-mediated vacuolar enlargement in post-embryonic root cell expansion, which previously could not be uncovered with embryonic-lethal *vti11/12* double mutants [11,96].

CRISPR/Cas9 is a powerful tool that will also be useful for studying cell-type-specific roles of vacuoles and cytoskeletons. A recent study developed an inducible cell-type specific CRISPR/Cas9-based genome editing system, allowing activation of the *Cas9* gene in cell types of interest under the control of an estrogen receptor-based gene transactivation system [97]. This technique has already been successful in conditionally knocking out key genes in specific cell types in roots, such as *WUSCHEL RELATED HOMEOBOX 5* (*WOX5*), *WOODEN LEG* (*WOL*), and *SCARECROW* (*SCR*) for the QC cells, vasculature, and endodermis or QC, respectively [97].

### 5.2. Strategies for Observing the Dynamics of Vacuoles and Cytoskeletons

Another obstacle that hampers research advances in this field is the difficulty of precisely determining the dynamics of vacuoles and cytoskeletons. Vacuoles have complex 3D structures, while cytoskeletons are dispersed as fine filaments within plant cells, and are dynamically reorganized at high speed. Rapidly evolving microscopy imaging is a promising method for detecting when, where and how plant cytoskeletons contact the vacuole and regulate its structure. The so-called super-resolution techniques, such as Structured Illumination Microscopy (SIM), Photoactivated Localization Microscopy (PALM), Stochastic Optical Reconstruction Microscopy (STORM), and Stimulated Emission Depletion microscopy (STED), generate images at higher resolution than traditional light microscopy [98]. Despite their limitations, e.g., difficult sample preparation and easy fluorescence bleaching, their high resolution at great depths have enabled them to be attractive means for visualizing cytoskeletal fibers. The dynamics of MTs in hypocotyls and roots have previously been revealed using the SIM and STORM techniques, respectively [99,100], while the STED technique has been employed to clarify the distribution of F-actin deep within *Arabidopsis* root epidermal cells [23]. Currently, such equipment is not readily available, but the capability of such techniques will greatly help elucidate the behavior of cytoskeletons in the vicinity of vacuolar surface.

Electron tomography is a technique used to reconstruct 3D structures of objects by transmission electron microscopy. Briefly, nanoscale 3D images are computationally generated using micrographs of the sample recorded from various orientations by tilting the specimen. The technique was recently used to successfully capture the long-debated process of vacuole biogenesis at nanometer resolution in *Arabidopsis* root cells [13]. The 3D images acquired by electron tomography have clearly captured connected and disconnected vacuoles, which previously could not easily be distinguished with conventional confocal microscopy, highlighting the overwhelming advantage of this technique [13]. Though not reported in plant research, this technique has already been applied to accurately determine the 3D network of MTs and F-actin in animal cells [101,102]. In the near future, it is expected that the nexus between vacuoles and cytoskeletons will be simultaneously visualized at extremely high magnification.

Combining these cutting-edge technologies could enable visualization of the native state of vacuoles and cytoskeletons in all plant cell types at the nanoscale and in 3D imaging formats.

## Data Availability

Not applicable.
